# Case Report: Stevens-Johnson syndrome following a single double dosing of nevirapine-containing regimen once in an HIV-infected woman on long-term antiretroviral therapy.

**DOI:** 10.12688/f1000research.6715.1

**Published:** 2015-06-30

**Authors:** Betty Kakande, Thuraya Isaacs, Rudzani Muloiwa, Sipho Dlamini, Rannakoe Lehloenya

**Affiliations:** 1 Division of Dermatology, Department of Medicine, University of Cape Town, Cape Town, 7700, South Africa; 2 Department of Paediatrics and Child Health, University of Cape Town, Cape Town, 7700, South Africa; 3 Division of Infectious Disease and HIV Medicine, Department of Medicine, University of Cape Town, Cape Town, 7700, South Africa

**Keywords:** HIV, nevirapine, double dose, Stevens-Johnson syndrome

## Abstract

A 31-year old HIV-infected African woman on nevirapine, tenofovir and lamivudine for more than 4 years presented with an 8-day history of symptoms and signs of Stevens-Johnson syndrome. She was on no other medication. Her viral load was undetectable and she had maintained a CD4 count of between 356 and 387cells/mm ^3^ in the preceding 2½ years. She missed her antiretrovirals 10 days before the onset of her symptoms and subsequently doubled her daily dose the following day. She had been on no other medication in the preceding 8 weeks.

Her ARVs were stopped and she fully re-epithelialized with the exception of the lips, over the following 10 days. She was started on a daily single tablet of Odimune® (a fixed drug combination antiretroviral containing tenofovir, emtricitabine and efavirenz).

Nevirapine is the most common offender in cases of antiretroviral-associated SJS in published literature. Lamivudine is very rarely implicated while there are no similar reports with tenofovir.  We concluded that nevirapine was by far the most likely offender in this case. Nevirapine toxicity is associated with high CD4 counts, undetectable viral load and high drug plasma level. We postulate that the sudden increase of the plasma levels of nevirapine in a patient with a high CD4 count and undetectable viral load created a perfect storm for the development of SJS in our patient, who had been on the NVP-containing regimen for many years.

Clinicians should be aware that severe adverse drug reactions are dynamic and can occur even when the drug has been in use for a long time.

## Background

Nevirapine (NVP)-based antiretroviral regimens have been used widely in developing countries because of its affordability, availability and efficacy ^1^. NVP is also used to minimize diarrhea and cardiovascular side effects of protease inhibitors and neuropsychiatric side effects of Efavirenz ^2^. However, NVP is associated with severe adverse reactions, including Stevens-Johnson syndrome (SJS).

In larger series, the incubation period before developing features of SJS ranges from 10–240 days with a median duration of around 12 days ^3^. We report a case of probable nevirapine-associated Stevens-Johnson syndrome occurring more than 4 years after initiation of the drug. This case illustrates that in susceptible persons, a severe drug reaction can occur when there are dose adjustments despite having been exposed to the same drug for a long time.

## Case report

A 31-year-old HIV-infected black African female presented with an 8-day history of painful swallowing, sore eyes, malaise and a worsening rash. She had been on antiretroviral (ARV) regimen of nevirapine (NVP) 400 mg daily, tenofovir 300 mg daily and lamivudine 300 mg daily for more than 4 years uneventfully. She was on no other medications and had not taken any other medication in the preceding 8 weeks. She had acquired HIV via heterosexual contact – the exact date of HIV infection was unknown. At the time of initiation of ARV therapy, her nadir CD4 cell count was 139 cells/mm ^3^ and her HIV RNA viral load at the time was unknown. Her last CD4 counts, done 31, 21 and 11 months before developing her current symptoms were 373, 356 and 387 cells/mm ^3^ respectively. The last HIV RNA viral load test, done 11 months prior to the onset of her symptoms showed an undetectable viral load.

She gave a history of forgetting to take her ARV medication for a day. The following day, 10 days before development of her symptoms she took 2 days equivalent of her ARVs in one day, in her own words “to make up for the missed dose”.

On examination, she was normotensive and had a temperature of 38.8°C. She had conjunctivitis and hemorrhagic cheilitis but no involvement of the genital mucosa. She had epidermal necrosis involving predominantly her trunk and face and to a lesser extent palms, soles and extremities, totaling 10% of her body surface area, 3% of which was stripping. Initial laboratory studies showed normal blood count, except for an elevated eosinophil count of 0.70 × 10 ^9^/L. The liver and renal function tests were normal. Her ARVs were stopped and her epidermal necrosis did not extend and eosinophil counts normalized. Over the next few days her skin condition improved and she was discharged 10 days later fully re-epithelialized with the exception of the lips, which were still eroded in areas. On review, 2 weeks after discharge, her skin had normalized except for residual hyperpigmentation. She was started on a single daily tablet of Odimune ^®^, a fixed drug combination of tenofovir 300 mg, emtricitabine 200 mg and efavirenz 600 mg. On follow-up, 4 weeks later she was tolerating the new ARV regimen.

## Discussion

Clinicians routinely blame NVP as the culprit agent in cases of ARV-associated SJS as it is by far the most common offender in published literature. However, lamivudine has infrequently been reported as a cause of SJS, but there have not been similar reports with tenofovir ^4^. Based on these, we concluded that NVP was by far the most likely offender in our case.

Numerous factors have been associated with NVP toxicity, including high current and nadir CD4 counts, undetectable viral load, female sex, abnormally elevated baseline transaminases, history of drug allergy, lower body weight and high drug plasma levels ^2, 5, 6^. We postulate that the sudden increase of the plasma levels of NVP in a patient with a high CD4 count and undetectable viral load created a perfect storm for the development of SJS in our patient, who had been on the NVP-containing regimen for many years. The impact of NVP drug concentrations on the risk of adverse drug reactions is still controversial. Some studies have shown that above a certain threshold, the risk of severe drug reactions is significantly increased. A recent study has shown that impaired renal function, thus impaired clearance of the drug, is associated with an odds ratio of 8.0 (3.9 to 17) to develop allopurinol-associated SJS in susceptible people ^7^. However other studies have not found this association ^6, 8, 9^. NVP has a long half-life, 25–30 hours, and the effect of doubling the dose possibly increased the plasma drug concentrations considerably resulting in SJS ^8^.

The patient had been compliant on treatment for more than 4 years and had undetectable viral load and a high CD4 count the last time they were measured, 11 months earlier. Both of these are independent predictors of NVP-associated SJS ^2, 10^. There were previous suggestions that the timing of SJS and other NVP-associated reactions coincided with the increase of the drug dose. However, this has been disproved, because as high as a third of cases in larger series occurred during the lead-in period of the dosing regimen before the dose was increased ^3, 11^. Pretreatment with ARVs longer than 1 year has also been reported as an independent predictor of a cutaneous drug reaction. Whether this is truly an independent predictor or a result of low HIV viral load or higher CD4 counts is unclear and further studies are needed to answer this question.

In summary, this case highlights the potentially life-threatening risk of the development of SJS outside the expected period, probably triggered by a spike in serum levels of NVP. The case also illustrates that development of severe drug eruptions, like SJS, is a dynamic processes that may evolve with changing environment in the body. Clinicians should warn their patients against unregulated dosing changes of their NVP-containing regimen.

## Consent

Written informed consent for publication of the clinical details was obtained from the patient.
