# Supplementary material for: Case Report: Stevens-Johnson syndrome following a single double dosing of nevirapine-containing regimen once in an HIV-infected woman on long-term antiretroviral therapy
Source: F1000Res. 2015 Jun 30;4:175. [Version 1] doi: 10.12688/f1000research.6715.1 (PMC4642844; doi:10.12688/f1000research.6715.1)
Supplement: Peer review history

# Peer review: Referee response for version 1

**DOI:** 10.5256/f1000research.7212.r10650

**Published:** 2015-11-06

**Authors:** Ronald Kiguba

**Affiliations:** 1 Department of Pharmacology and Therapeutics, College of Health Sciences, Makerere University, Kampala, Uganda

The authors highlight an important report of Stevens-Johnson syndrome (SJS) in a patient on nevirapine-based antiretroviral therapy for more than two years. The median duration to the onset of  nevirapine-associated SJS is two weeks, thus the case report stimulates exploration of the relationship between sudden rise in plasma nevirapine concentration and increased risk of SJS. However, it was not clear whether the authors performed any formal adverse drug reaction causality assessment, even though they stated that the reaction was "probable".

I have read this submission. I believe that I have an appropriate level of expertise to confirm that it is of an acceptable scientific standard, however I have significant reservations, as outlined above.

# Peer review: Referee response for version 1

**DOI:** 10.5256/f1000research.7212.r10651

**Published:** 2015-10-20

**Authors:** Carla Ferrándiz-Pulido

**Affiliations:** 1 Department of Dermatology, Vall d'Hebron University Hospital, Barcelona, Spain

This is an interesting case of Stevens-Johnson syndrome probably due to a single double dosing of nevirapine-containing regimen in an HIV-infected woman. There have been a lot of case reports and short series of SJS/TEN due to antiretroviral drugs. However, the singularity of  this case is that the drug had been taken for a long time (4 years), and the triggering was an increase in the dosage. This side effect due to an increase in the dosage has also been reported for allopurinol.

Most of Lyell's syndrome/TEN are caused by drugs; however, SJS may be triggered by a "new" drug or also an infection such as VHS/VVZ or *Mycoplasma pneumoniae*. In my opinion, it is always important to rule out such infections, when a SJS in suspected. This should be included in the article.

Some clinical images of the skin and mucosal involvement would improve the quality of the article.

I have read this submission. I believe that I have an appropriate level of expertise to confirm that it is of an acceptable scientific standard, however I have significant reservations, as outlined above.

# Peer review: Referee response for version 1

**DOI:** 10.5256/f1000research.7212.r9555

**Published:** 2015-07-20

**Authors:** Neil Shear

**Affiliations:** 1 Medical School, University of Toronto, Toronto, ON, Canada

The Title, Abstract and all content of the article are clear and relevant.

This case could seem to be simple but it actually adds a valuable perspective to our understanding of what might trigger severe drug reactions and adds clinical insights and clarity to what is often an overly complex environment.

I have read this submission. I believe that I have an appropriate level of expertise to confirm that it is of an acceptable scientific standard.
